# Quantitative PCR for etiologic diagnosis of methicillin-resistant *Staphylococcus aureus* pneumonia in intensive care unit

**DOI:** 10.1186/1753-6561-5-S6-P66

**Published:** 2011-06-29

**Authors:** T Jeon, D Seo, SJ Kwon, J Kang

**Affiliations:** 1 Medical School of Konyang, Konyang University, Daejeon, Korea, Republic Of; 2 Department of Internal Medicine, Konyang University Hospital, Korea, Republic Of; 3 College of Medicine, Konyang University, Daejeon, Korea, Republic Of

## Introduction / objectives

Because methicillin-resistant *Staphylococcus aureus* (MRSA) was frequent pathogen in ventilator-associated pneumonia (VAP), the rapid identification of it from respiratory samples was important. Therefore, our aim was to evaluate the utility of qPCR as a useful method for etiologic diagnoses of MRSA-based pneumonia.

## Methods

We performed qPCR for mecA, *S. aureus*-specific femA-SA and *S. epiderdimis*-specific femA-SE genes from bronchoalvolar lavage (BAL) or bronchial washing samples obtained from clinical suspected VAP. We spiked an internal control (SPUD) at in the course of DNA extraction. We estimated colony forming units (CFU/ml) of MRSA samples through a standard curve of a serially diluted reference MRSA strain. We compared threshold cycle (Ct) value of MRSA of clinical samples with the microbiologic culture results of that.

## Results

We examined 72 samples of 64 patients with clinical suspected VAP. We obtained the mecA gene standard curve. It showed that the detection limit of the mecA gene was 100fg, which corresponded to a copy number of 30. We chose cut-off Ct values of 27.94 (equivalent to 1 × 10^4^ CFU/ml) and 21.78 (equivalent to 1 × 10^5^ CFU/ml). Using these cut-off values, the sensitivity and specificity of our assay was 88.9% and 88.9%, respectively, when compared with quantitative cultures.

## Conclusion

Our results were valuable for diagnosing and identifying pathogen for VAP. We suggest that our modified qPCR method is an appropriate and rapid tool for diagnosing of clinical pathogens in intensive care unit (ICU) patients.

## Disclosure of interest

None declared.

